# An Innovative Technique of Reconstructing Combined Extensor Tendon Zone (z5 and z4) Defect, Utilizing an Adjacent Proximal (z6) Tendon, as a Distally Based Tendon Flip Flap, in the Index Finger

**DOI:** 10.1055/s-0045-1802316

**Published:** 2025-01-28

**Authors:** Narendra S. Mashalkar, Pooja Thoppal Shiva, Joseph Kattady

**Affiliations:** 1 Department of Plastic Surgery and Burns, St John's Medical College, Bangalore, Karnataka, India

**Keywords:** combined extensor tendon defect, tendon flip flap, index finger

## Abstract

Delayed reconstruction of segmental extensor tendon defects of the hand has been described with auto-tendon grafting or by using local flaps harvested from the native tendon. These reconstructive techniques have been described in certain extensor tendon zones, mainly in zones 2, 4, and 5 of the hand. Borrowing auto-tendon grafts will have donor site morbidity. The use of available local tendon as flap may be considered, the tendon harvesting being done from a part of the native tendon from either proximal or distal location. The index and little fingers have the advantage of having two tendons to do the same function and hence these tendons may be utilized as a whole to our advantage as distally based tendon flip flap to reconstruct zone 4 and 5 extensor tendon defects with good functional outcomes.

## Introduction

Segmental extensor tendon defects of the hand, which cannot be repaired primarily, needs either an auto-tendon graft or a part of tendon harvested from either the distal or the proximal part of the native, uninjured part of the tendon to bridge the tendon defect. The auto-tendon graft has been harvested from the palmaris longus, the tensor fascia lata, or the plantaris. A literature search on surgical management of extensor tendon defects in the respective zones in the hand reveals that these are managed either by auto-tendon graft or by local tendon flaps. The anatomy of zone 5 is more complex, with an extensor hood being the main part. There is also paucity of literature in surgical management of zone 5 extensor tendon defect in utilizing extensor indicis proprius (EIP) tendon as a flap. We present here a simple method of managing zone 5 extensor tendon defect, extending into zone 4, with loss of extensor hood. This technique utilizes the adjacent EIP tendon as a distally based tendon flap, which is flipped and bridged to reconstruct the extensor digitorum communis (EDC) tendon defect. The bridged tendon acts as the hood.

## Case History



**Video 1** Intraoperative tendon flap harvest.


**Fig. 1:**
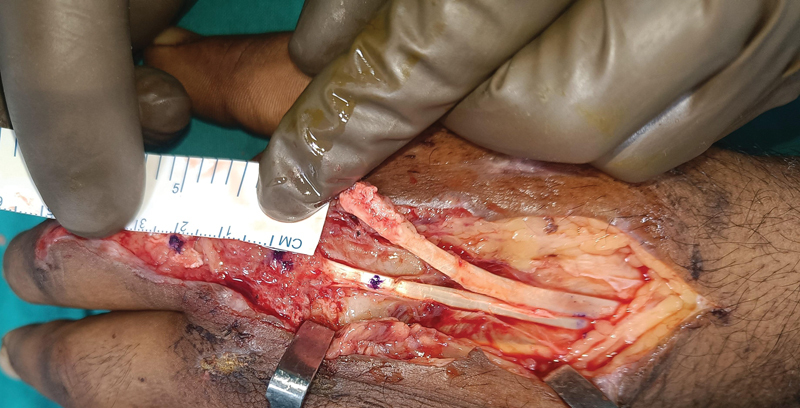
Tendon defect measurement.

**Fig. 2:**
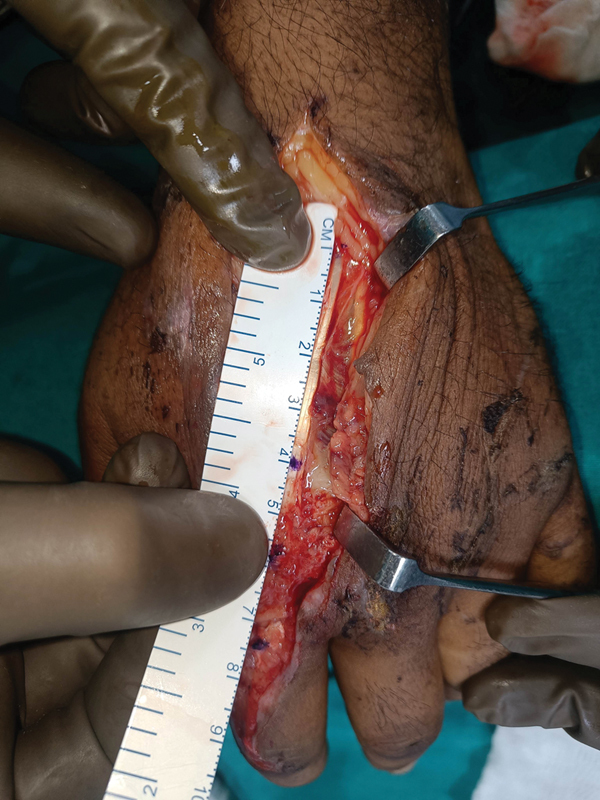
Intraoperative image of extensor indicis proprius (EIP) tendon measurement.

**Fig. 3:**
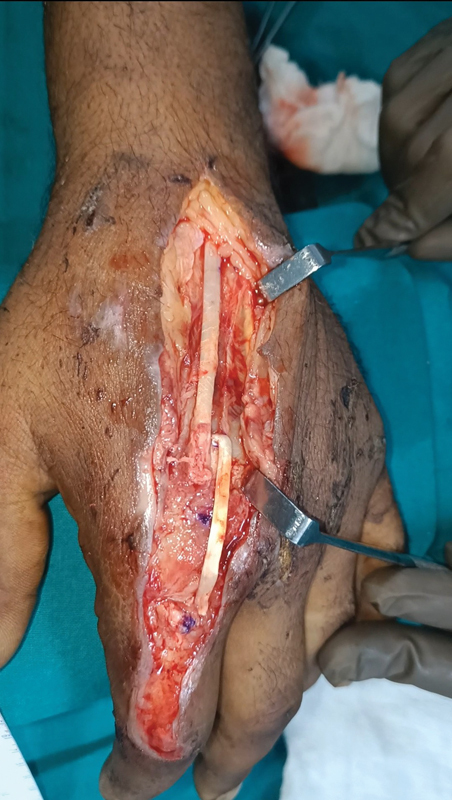
Extensor indicis proprius (EIP) tendon flap being flipped.

**Fig. 4:**
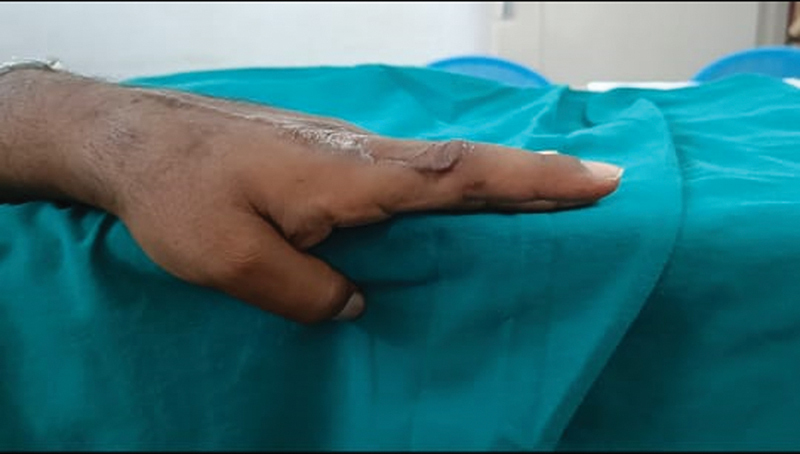
Post-op metacarpophalangeal (MP) in extension.

A 45-year-old gentleman, driver by occupation, met with accidental blunt injury in the abdomen with composite soft tissue injury over the dorsum of the metacarpophalangeal (MP) joint of the left index finger without any underlying bone fracture. He was treated for a blunt abdomen injury and was referred to us after 3 weeks for treatment of the hand injury. He had a zone 5 extensor tendon defect, extending distally to a part of zone 4, involving both the EIP and EDC to the index finger with loss of the extensor hood. The tendon defect was 2 cm ( Fig. 1 ), with the MP joint in the neutral position. The EIP tendon was exposed proximally in zone 6, and 4-cm-long proximal EIP ( Fig. 2 ) was harvested as a distally based flap ( Fig. 3 ), with 2 cm being the base of tendon flap and flipped. The base of the EIP tendon is firmly fixed to its native soft tissue. This part was not disturbed and there was neither turning nor shearing seen while weaving and handling the tendon flap suturing. Video 1 describes the harvest of the EIP tendon. Of the total length of the EIP tendon exposed, 4 cm of the EIP tendon was harvested, with the base length being 2 cm, and 2 cm of the harvested tendon was used to bridge the gap. With dissection and mobilization of the distal cut end of the tendon, with the MP joint in extension, the rest of the tendon flap was repaired by a single weave with double breasting. The proximal cut end of the EDC tendon was sutured to the base of the EIP tendon flap by simple suturing and this reinforced the base of the flap, preventing avulsion from its native tissue. The tip of the EIP tendon flap, that is, the proximal end of the EIP tendon, was weaved and sutured to the distal end of the EDC tendon at zone 4. After the weave and tendon repair, there was a soft tissue defect (1 cm × 1 cm) on the dorsum of the MP joint, which was covered by a proximal based local transposition flap harvested from the dorso-ulnar side. The incision on the ulnar border of the marked flap did not cross the glabrous region of the finger. Hence, we did not visualize the ulnar neurovascular bundle. The secondary defect was covered by a split-thickness skin graft. A slab was applied with the wrist in the neutral position, MP joint in 10-degree extension, and the proximal interphalangeal joint (PIP) and distal interphalangeal joint in the neutral position for 4 weeks. Physical therapy in the form of gentle, sustained active movements was begun at week 5. At 10 weeks postoperatively, the patient had developed full range of active extension at the MP joint ( Fig. 4 ).

## Discussion



**Video 2** Post-op range of movements at the MP joint.


**Fig. 5:**
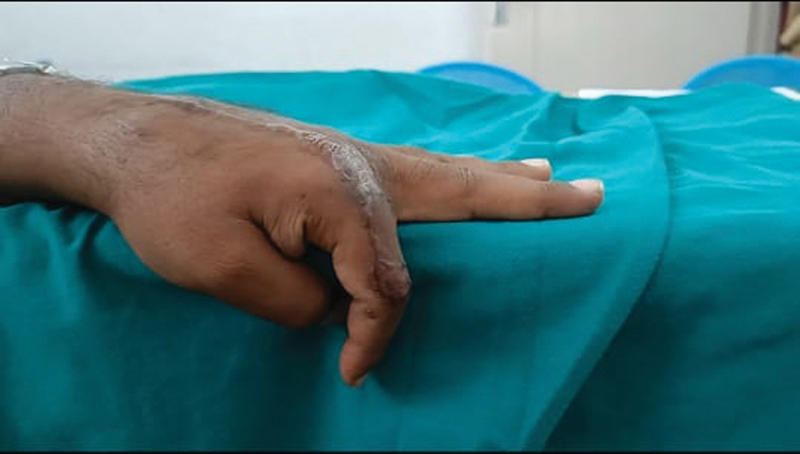
Post-op metacarpophalangeal (MP) in flexion.

The extensor tendon of the hand has seven anatomical zones, 1 and injury to each anatomical zone with a tendon defect may be reconstructed by a free tendon auto-graft 2 to bridge the defect or by a local tendon flap. 3 At the index and little fingers, there may be two tendons 4 performing the same function; hence, one of the adjacent tendons may be used as a tendon flap. Our case had an index finger EDC and EIP tendon defect of 2 cm at zone 5 encroaching on to zone 4. Hence, the EIP tendon was used as a distally based tendon flap to bridge the defect. Cerovac and Miranda had a zone 4, 1-cm defect of the EIP and EDC tendons, reconstructed from the inner halves of the tendon by elongating the tendon in an **L** -shaped incision. 5 Kochevar et al 3 have described local tendon flaps to reconstruct a tendon defect of 0.5 cm in zones 2 and 4. However, the intricacies or surgical details have not been described. We have used a distally based EIP tendon flap of approximately 4 cm to flip and bridge the zone 5 defect. Carroll et al have described reconstruction of a collateral band in zone 5 using a distally based part of an ulnar tendon loop. 6 7 Our technique involved single end weaving with double breasting tendon repair. This was done to give the holding strength of tendon suture, with the distal end of the suture being done at zone 4. Willkomm et al have also mentioned adequate overlap of the tendon while suturing to give strength. 7 We performed the EIP tendon weaving and suturing in the proximal end of zone 4. Hence, the bridged EIP tendon acted as extensor hood, which would facilitate the gliding of the tendon during movement at the MP joint. In our case, at 10 weeks post-op ( Video 2 ), the range of motion showed flexion of 90 degrees with no extensor lag at the MP joint and 90-degree flexion with no extensor lag at the PIP joint ( Fig. 5 ). Turker noted that the extensor tendons in zones 1 to 5 have minimal excursion; hence, even a 1-mm loss of extensor tendon substance will lead to a loss of 20 degrees of extension at the PIP joint, with loss of finger flexion. 8

In our case, flexion of the PIP joint could be achieved only till 90 degrees; hence, we concur with the observation made by Turker. Cerovac and Miranda 5 have also noted zero extensor lag at the MP joint with 70 degrees of flexion and zero extensor lag at the PIP joint with 65 degrees of flexion.

## Conclusion

EDC tendon reconstruction with absent extensor hood, using the EIP tendon as distally based tendon flip flap at the index finger zone 5, has given good extension at the MP joint without any extensor lag. There was also no radial or ulnar slide of the tendon, and adequate flexion was observed at the PIP joint without any extensor lag. This innovative method of reconstruction of the index finger zone 4 and 5 tendon defect is simple and takes advantage of the adjacent additional tendon. When an adjacent extra tendon exists, which performs a similar function, then its utilization as a local flap to perform extension with hood function would give good functional outcome without donor site morbidity.
